# Peri-Implant Mucosa Augmentation with an Acellular Collagen Matrix

**DOI:** 10.3390/membranes11090698

**Published:** 2021-09-12

**Authors:** Gregor-Georg Zafiropoulos, Adel A. Al-Asfour, Moosa Abuzayeda, Zeljka Perić Kačarević, Colin Alexander Murray, Branko Trajkovski

**Affiliations:** 1 Department of Surgical Sciences, Faculty of Dentistry, Kuwait University, 13110 Safat, Kuwait; ggzafi@gmx.de (G.-G.Z.); adel.alasfour@ku.edu.kw (A.A.A.-A.); 2 Department of Prosthodontics, College of Dentistry, MBR University, Dubai 505055, United Arab Emirates; Moosa.Abuzayda@mbru.ac.ae; 3 Department of Anatomy Histology, Embryology, Pathology Anatomy and Pathology Histology, Faculty of Dental Medicine and Health, University of Osijek, 31000 Osijek, Croatia; zeljkapericc@gmail.com; 4 Department of Preventive and Restorative Dentistry, College of Dental Medicine, University of Sharjah, Sharjah 27272, United Arab Emirates; profcolinmurray@outlook.com

**Keywords:** collagen, dental implants, collagen matrix, soft tissue augmentation, keratinized mucosa

## Abstract

Peri-implant keratinized mucosa (PI-KM) may support implant survival. Acellular collagen matrices (aCMs) have been widely used to facilitate soft tissue regeneration. The aim of this study was to investigate clinical outcomes obtained with the use of an aCM (mucoderm^®^) to enhance PI-KM. In this retrospective non-randomized case series, 27 restored implants in 14 patients (eight males and six females, mean age = 56 years) with a PI-KM width ≤ 1 mm were followed for 6 months. It was demonstrated that aCM grafts augmented PI-KM effectively (mean increase of 5.4 mm; >533%) without a significant change in bleeding on probing (BOP) from baseline. The mean aCM shrinkage was 3.9 mm (42%). Gender, area, arch, and BOP did not influence PI-KM augmentation or aCM shrinkage significantly. The present results demonstrated that the examined aCM was effective and predictable for attaining a band of keratinized tissue, while avoiding graft donor site harversting.

## 1. Introduction

The use of dental implants is an effective procedure to replace missing teeth, with implant survival rates up to 95% after 10 years of prosthetic loading [1,2]. There is ongoing debate regarding the importance of keratinized tissue width for maintenance of periodontal and prevention of soft tissue recession, with some recommending a minimum width of 2 mm for maintenance gingival health [3,4]. However, provided that traumatic tooth brushing and inflammation are controlled, patients have been reported to maintain gingival health without deterioration or progression of gingival recession with a minimal band of gingiva [3,4]. Thus, a narrow band of attached keratinized gingiva alone is not considered an absolute indication for gingival augmentation.

Nevertheless, implant sites with < 2 mm of keratinized mucosa (KM) have been found to be prone to brushing discomfort, plaque accumulation, and peri-implant inflammation compared to implant sites with more keratinized tissue [5,6]. Implant failures can be classified as mechanical or biologic, with peri-implant diseases being considered as the most common causes of implant-related biologic complications [7]. Although peri-implant keratinized mucosa (PI-KM) has not been established as a prognostic factor for implant survival [8], the presence of PI-KM has been associated with implant-neck seal stability that facilitates cleaning and limiting bacterial infiltration into the rehabilitated region [7,9]. The width and thickness of PI-KM do not appear to compromise the mechanical stability of implants. However, they have been correlated negatively with peri-implant soft-tissue recession, plaque accumulation and inflammation [3]. Moreover, a narrow band of PI-KM together with a technical complication such as loosening of the abutment screw could lead to fistula formation and subsequent peri-implantitis and implant failure [8,10].

Given the common view of KM width as a keystone for the maintenance of periodontal and peri-implant health, free gingival graft (FGG) placement to increase KM width has become common [4,11,12,13,14,15,16]. However, areas augmented by FGGs are characterized by differences in texture and color from those of adjacent soft tissues. Furthermore, FGGs must be harvested from palatal donor sites, which entails postoperative morbidity and limited availability [6,11,12,13,17]. Collagen matrices (CMs) have been introduced as an alternative materials for soft-tissue augmentation, but few clinical studies have examined the use of CMs for PI-KM width augmentation [18,19,20,21,22]. In particular, there are not enough data to conclude whether CMs increase PI-KM width effectively in implant areas.

In the present study, an acellular collagen matrix (aCM) was used as a grafting material to increase the PI-KM width and to avoid donor-site harvesting. Therefore, we conducted a retrospective analysis of 27 implant restorations in which the PI-KM was treated with the aCM during a 6 month observation period.

## 2. Materials and Methods

### 2.1. Study Population

**Figure 1:**
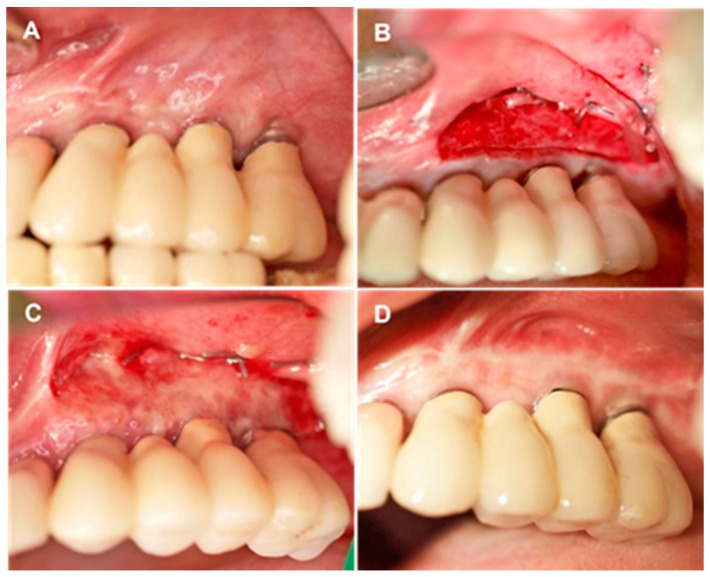
Overview of treatment stages. (**A**) Initial clinical view of area encompassing sites #23 to #26 after implant loading. PI-KM width = 1 mm. (**B**) Intraoperative view of aCM placement and fixation on the periosteum. (**C**,**D**) Soft tissue healing in area encompassing sites #23 to #26, 10 days postoperatively (**C**) and 6 months postoperatively (**D**).

In this retrospective non-randomized case series, 27 prosthetically restored implants in 14 patients (eight males and six females, mean age = 56 years) were analyzed. In the target areas, only a small amount of PI-KM was observed. The patients were treated by one of the authors (GGZ) between 2012 and 2016. Only one area per patient (randomly selected if more than one) was included in the present analysis. The inclusion criteria were: (1) non-smoker; (2) good systemic and periodontal health; (3) PI-KM width ≤ 1 mm in target implant area (Figure 1A); (4) implant restorations loaded ≥3 months prior to the present treatment; (5) already enrolled in a supportive periodontal care program consisting of three follow-up appointments per year, demonstrating good oral hygiene and compliance (full mouth plaque score ≤ 12%); and (6) willingness and ability to provide informed consent.

### 2.2. Surgical Treatment and Clinical Measurements

Alveolar mucosa was stained with Schiller iodine solution to facilitate mucogingival junction identification. Local anesthesia was achieved with 4% articaine HCl injection containing 1:100,000 epinephrine (Ultracain D-S forte; Sanofi-Aventis, Frankfurt/M, Germany). A horizontal mucosal incision was made in the mucogingival junction with a #15 surgical blade and extended for 3 mm from the mesial level of the first implant and distal to the last implant. A partial thickness mucosal flap was prepared, displaced apically, and sutured at the base of the newly created vestibule with 5-0 non-resorbable sutures (Ethibon; Ethicon-Johnson & Johnson, Cincinnati, OH). Coronal PI-KM was not removed or retracted (Figure 1B).

A three-dimensional 1.7 mm-thick porcine derived aCM (mucoderm^®^; 30 × 40 mm^2^ or 15 × 20 mm^2^; EC 0483; botiss biomaterials, Zossen, Germany) was used for soft tissue augmentation. The aCM was hydrated in sterile saline solution for 10 min, trimmed, positioned in the vestibule, and fixated on the periosteum with interrupted simple-loop resorbable sutures (5-0 Monocryl; Johnson & Johnson Int., Neuss, Germany). The fixated aCM was left exposed, and moist gauze was applied to the grafted site with light finger pressure for 5 min to minimize blood clot formation between the aCM and surrounding tissues (Figure 1B).

The following clinical parameters of target areas were assessed with a periodontal probe (PCPUNC156; Hu-Friedy, Frankfurt/M, Germany) and rounded to the nearest half millimeter perioperatively (T0) and/or 6 months after surgery (T1): width of aCM used (T0); PI-KM width from the buccal margin to the mucogingival junction (T0, T1); and bleeding on probing (BOP) at the buccal site of the peri-implant pocket (T0, T1).

### 2.3. Postoperative Care

Patients were instructed on how to brush around the grafted site with a soft toothbrush and told to rinse with only water, using no chlorhexidine or any other mouth solution, during the healing period. They were given a prescription for an anti-inflammatory (ibuprofen, 600 mg) and given instructions on its as-needed use for pain and swelling. The non-resorbable sutures were removed 10 days after the surgical procedure (Figure 1C). Patients were scheduled for oral hygiene maintenance, including weekly supragingival cleaning and polishing for the first five postoperative weeks, and then enrolled in supportive periodontal care (Figure 1D).

### 2.4. Data Analysis

Demographic variables were reported at the patient level (one measurement per patient), and implant variables at the implant level. Continuous variables were reported as means with standard deviations (SDs) and ranges; categorical variables were reported as numbers and percentages. The Wilcoxon matched-pairs test was used to evaluate changes in PI-KM from T0 to T1, as well as to evaluate the percentage change. Descriptive statistics of aCM shrinkage were recorded on metric (Equation (1)) and percentage (Equation (2)) scales as follows:

aCM shrinkage (mm) = (PI-KM T0 + aCM T0) − PI-KM T1(1)

aCM shrinkage (%) = aCM shrinkage (mm) / aCM T0 × 100(2)

Additional analyses examined how shrinkage of aCM (in mm) and change in PI-KM (in mm) varied between patient subgroups, such as by gender or arch). The Mann–Whitney test was used for these analyses. The cut-off for statistical significance was *p* ≤ 0.05 in all cases. Statistical analyses were conducted in Stata software (v. 15.1; StataCorp LLC, College Station, TX).

## 3. Results

Demographic characteristics of the patient sample and their implant locations are reported in Table 1. Briefly, the patients’ ages spanned a 29 year range, and there were similar numbers of men and women. Two-thirds of implants were placed in a posterior area, and almost two-thirds in the maxillary arch.

The results of study outcome variables, including changes from T0 to T1 and associated p values, are reported in Table 2, Table 3 and Table 4. Analyses were performed to summarize the outcomes and to examine changes in outcomes from T0 to T1. The results for the width of PI-KM are summarized in Table 2. Across 27 implants, the width of PI-KM increased from T0 (1.1 ± 0.3 mm) to T1 (6.5 ± 0.9 mm). This difference (5.4 mm on average, or 533% change) was statistically significant (*p* < 0.001; Table 2).

Further analyses examined whether aCM shrinkage varied between different groups. A summary of the results is displayed in Table 3. The results suggested that there was no significant difference in shrinkage between any of the groups. Shrinkage did not vary by gender, area, arch, or BOP. The mean mCM shrinkage was 3.9 mm. On the percentage scale, the mean value was 42%.

## 4. Discussion

In this study, the postoperative healing and maintenance period were uneventful, with no complications of implant loss or severe inflammation.

Despite the substantial aCM shrinkage from T0 to T1 (mean shrinkage of 3.9 mm (42%)), significant PI-KM width augmentation was observed 6 months postoperatively (mean increase 5.4 mm, mean percentage increase of 533%). More specifically, no significant differences in shrinkage were found in relation to gender, area, arch, or BOP. In comparison to our results, Papi et al. observed a mean increase in the PI-KM width of 2 mm after the use of the aCM and Sanz et al. reported a mean KM width increase of 2.5 mm with the use of a different CM [17,18,20]. The shrinkage observed in our study falls within the range of reported FGG shrinkage (17–58%), depending on smoking habit and/or tissue phenotype [4,14,15,16,23]. Sanz et al. used a CM Mucograft^®^ as an alternative to FGG and reported a shrinkage of 67% [17]. This might be due to variations in the collagen production process, which can alter clinical outcomes [24,25].

The aCM used in this study is processed without chemical cross linking, as the antigenic cellular components are removed while preserving the structural integrity [26]. Previous histological analysis has revealed that it integrates well, with no foreign body reaction [27]. In fact, the aCM proved to be an excellent scaffold for the formation of new connective tissue [27]. In an animal model, the rough and porous collagen structure of this material was determined to serve as a scaffold for blood vessel and cell ingrowth, enabling revascularization of the matrix and integration of the aCM into the surrounding tissue with progressive complete remodeling [28,29]. Additionally, the FGGs and aCM had comparable result in vestibuloplasty leading to desirable PI-KM sufficiency, although the integration of FGGs (revascularization) and aCM (new tissue formation) differ biologically [30].

The results of this study are not conclusive, due mostly to the limitations associated with our small sample and lack of a control group. However, they suggest that the range of aCM shrinkage observed in this study cannot be attributed to demographic factors or restoration location. Variance in shrinkage may reflect inter-individual differences not examined in this study, such as matrix exposure in the oral cavity, nutritional factors, microbe presence or salivary components. Given the limited information available, we recommend that clinicians ensure close contact between the aCM and underlying periosteum, combined with secure fixation to prevent micro-movements that could destroy newly formed blood vessel networks. It would be interesting to check if the use of additional biomaterials in combination with aCM will provide enhanced regenerative effect [31].

## 5. Conclusions

Within the limitations of this study, the present clinical case series demonstrated that the PI-KM width can be increased effectively with aCM use. The avoidance of tissue harvesting from the palate leads to minor postoperative discomfort. Further studies with a larger number of treated test and control sites are necessary to verify these findings.

## Figures and Tables

**Table 1:** Patient demographics and implant sites (mean ± standard deviation, range, or N (%).

Measurement Level	Variable	Category	Summary
Patients (*n* = 13)	Age	-	53.4 ± 8.3 (39, 68)
	Gender	Female	7 (54%)
		Male	6 (46%)
Implants (*n* = 27)	Area	Posterior	18 (67%)
		Anterior	9 (33%)
	Arch	Maxilla	17 (63%)
		Mandible	10 (37%)

**Table 2:** Summary of outcomes and changes from T0 to T1 (Width PI-KM only).

Variable	Timepoint—Statistic	*n*	Summary	*p*-Value
Width PI-KM (mm)	Time 0—Mean ± SD	27	1.1 ± 0.3	
	Time 1—Mean ± SD	27	6.5 ± 0.9	
	Change ^(*)^—Mean (95% CI)	27	5.4 (5.0, 5.7)	<**0.001**
	% Change ^(**)^—Mean (95% CI)	27	533 (450, 616)	<**0.001**

(*) Change calculated values at T1 minus value at T0. (**) % change calculated as values at (T1–T0)/Time 0 × 100.

**Table 3:** Group comparisons for aCM shrinkage (mm).

Variable	Category	*n*	mCM shrinkage. Mean ± SD	*p*-Value
Gender	Female	13	3.7 ± 1.4	0.81
	Male	14	4.1 ± 0.9	
Area	Posterior	18	3.9 ± 1.1	0.57
	Anterior	9	3.9 ± 1.3	
Arch	Maxilla	17	3.9 ± 1.2	0.74
	Mandible	10	3.9 ± 1.1	
BOP (Time 0)	Negative	18	3.9 ± 1.1	0.84
	Positive	8	3.8 ± 1.3	

**Table 4:** Group comparisons for change in PI-KM (mm).

Variable	Category	*n*	PI-KM change Mean ± SD	*p*-Value
Gender	Female	13	5.5 ± 1.0	0.69
	Male	14	5.3 ± 0.9	
Area	Posterior	18	5.4 ± 0.9	0.83
	Anterior	9	5.3 ± 1.0	
Arch	Maxilla	17	5.5 ± 0.9	0.21
	Mandible	10	5.1 ± 0.9	
